# Two base pair deletion in IL2 receptor γ gene in NOD/SCID mice induces a highly severe immunodeficiency

**DOI:** 10.1186/s42826-020-00048-y

**Published:** 2020-08-14

**Authors:** Inseon Bak, Doo-Jin Kim, Hyoung-Chin Kim, Hye-Jun Shin, Eunhye Yu, Kyeong-Won Yoo, Dae-Yeul Yu

**Affiliations:** 1 Korea Research Institute of Bioscience and Biotechnology (KRIBB), 125 Gwahak-ro, Yuseong-gu, Daejeon, 34141 Korea; 2 Genome engineering laboratory, GHBIO Inc., C406, 17 Techno4-ro Yuseong-gu, Daejeon, 34013 Korea; 3 Korea Research Institute of Bioscience and Biotechnology (KRIBB), 30 Yeongudanji-ro, Ochang-eup, Cheongwon-gu, Cheongju, Chungcheongbukdo 28116 Korea

**Keywords:** CRISPR/Cas9, IL2 receptor γ, Small deletions, Immunodeficient mouse

## Abstract

Genome editing has recently emerged as a powerful tool for generating mutant mice. Small deletions of nucleotides in the target genes are frequently found in CRISPR/Cas9 mediated mutant mice. However, there are very few reports analyzing the phenotypes in small deleted mutant mice generated by CRISPR/Cas9. In this study, we generated a mutant by microinjecting sgRNAs targeting the IL2 receptor γ gene and Cas9 protein, into the cytoplasm of IVF-derived NOD.CB17/Prkdcscid/JKrb (NOD/SCID) mice embryos, and further investigated whether a 2 bp deletion of the IL2 receptor γ gene affects severe deficiency of immune cells as seen in NOD/LtSz-scid IL2 receptor γ^−/−^ (NSG) mice. Our results show that the thymus weight of mutant mice is significantly less than that of NOD/SCID mice, whereas the spleen weight was marginally less. T and B cells in the mutant mice were severely deficient, and NK cells were almost absent. In addition, tumor growth was exceedingly increased in the mutant mice transplanted with HepG2, Raji and A549 cells, but not in nude and NOD/SCID mice. These results suggest that the NOD/SCID mice with deletion of 2 bp in the IL2 receptor γ gene shows same phenotype as NSG mice. Taken together, our data indicates that small deletions by genome editing is sufficient to generate null mutant mice.

## Introduction

The rodent model is a useful tool for in vivo studies related to gene functions, mechanisms of disease, and new therapeutic agents. However, translation of these studies is limited due to species differences between rodents and humans. Non-human primates are the best alternative model of human disease, but extremely high costs and ethical concerns have limited their widespread use. Recently, humanized mice have been developed and used to replace primates. Immunodeficient mice deficient in T, B and natural killer (NK) cells are accepted as recipients for human cell transplant and have been widely used in human disease studies, including human-specific infections such as viral infections (HIV, EBV, dengue virus, HBV, HCV) [1, 2] and bacterial infections. These mice have been used to study regenerative medicine such as allograft, organ regeneration, and drug evaluation [3–6].

Interleukin 2 (IL2), also known as the T-cell growth factor, has a broad functional role in the growth and activation of B cells, NK cells, and macrophages [7, 8]. The action of IL-2 is mediated through the IL2 receptor (IL2R) which comprises of α, ß, and γ chains. As observed by flow cytometric analyses, the γ chain is expressed on almost all cell populations [9, 10]. Of these, the Interleukin-2 receptor γ (IL2Rg) chain is known as the common cytokine receptor γ-chain and is the most crucial component of the receptors for IL2, − 4, − 7, − 9, and − 15 [11, 12]. Patients devoid of IL2 have normal numbers of T cells, but patients lacking IL2Rg are deficient of T cells. Since the γ chain is shared among the multiple cytokine receptors, mutation of IL2Rg in mice impaired the development and function of T, B and NK cells [13, 14]. The immunodeficient NOD/SCID mice are important models for investigating carcinogenesis, cancer therapy, and imaging of tumor growth and metastasis [15]. The major immunodeficient models are NOD/LtSz-scid IL2Rg^−/−^ (often referred to as NSG mice) and NOD/Shi-scid IL2Rg^−/−^ (often referred to as NOG mice). These models are generated by breeding between NOD/Shi-scid mice and IL2Rg null mice, and have shown superiority in human cancer xenografts, as compared to nude mice [16].

Genome editing using programmable nucleases such as zinc finger nuclease (ZFN), transcription activator-like effector nucleases (TALENs), and CRISPR/Cas9 nucleases, is an efficient strategy to generate genetically engineered mice (GEM) [17]. Particularly, the CRISPR/Cas9 technology is the most convenient method to produce GEM, owing to its simple construction and high DNA double-strand break (DSB)-inducing activity [3]. A CRISPR/Cas9 system consists of Cas9 nuclease and a single guide RNA (sgRNA) that targets a specified genomic locus defined by the sgRNA and a protospacer adjacent motif (PAM) sequence; this system cleaves the double-stranded DNA. The DSBs are repaired by error-prone non-homologous end-joining (NHEJ), randomly inducing insertions or deletions, which result in targeted gene disruption.

To generate knockout mice using the CRISPR/Cas9 system, Cas9 and sgRNA used for microinjection are generally synthesized using in vitro methods [18–20]. To generate knockout mice with genome editing technology, fertilized embryos are generated by crossing male and super-ovulated females. However, since it is difficult to generate fresh fertilized eggs in NOD/SCID mice [21], in vitro fertilization (IVF) procedures are applied to produce the embryos. It has recently been reported that freshly fertilized oocytes produced by IVF can be used for conventional CRISPR/Cas9 genome editing [22]. Therefore, we applied IVF technology and the CRISPR system to produce the NOD/SCID mice deleted IL2Rg gene (referred as NIG or NSIG), and analyzed if a small deletion in a mutant can induce the same phenotype as knockout mice generated by gene targeting.

## Materials and methods

### IL2Rg sgRNAs synthesis

Targeting sequence constituted of 20 nucleotides followed by an “NGG” sequence called PAM. For RNA Transcription, Template DNA was prepared by annealing each pair of oligonucleotides: Mix 10 μl each of 100 μM primers and cloning into a pT7-gRNA vector. Plasmid vectors were digested with a restriction endonuclease (BamH1) for overnight at 37 °C and purified by phenol:chloroform extraction and isopropanol precipitation. Guide RNAs were synthesized using MEGAshortscript T7 Transcription Kit (Ambion, USA) according to the manufacturer’s instructions. For microinjection, synthesized sgRNAs were purified by phenol:chloroform extraction and isopropanol precipitation.

### Ethics statement

All mice were maintained in individually ventilated cages (IVC) located in a specific pathogen-free (SPF) room at a constant temperature of 22 ± 1 °C, humidity of 55 ± 10%, and 12 h light/dark cycle. All animal procedures were executed in accordance with the guidelines of the Institutional Animal Care and Use Committee, Korea Research Institute of Bioscience and Biotechnology (KHMC-IACUC-16015).

### Superovulation and IVF

NOD.CB17/Prkdcscid/JKrb (NOD/SCID) mice have been bred in KRIBB for more than 20 years after receiving them from Jackson Laboratory in 1999. Female NOD/SCID mice (4–5 weeks of age) were superovulated by intraperitoneal injection with 5 IU pregnant mare serum gonadotropin (sigma, St. Louis, USA), followed 46 h later by injection of 5 IU human chorionic gonadotropin (hCG, sigma, USA). The animals were sacrificed 14 h following hCG administration and oviducts were collected. Cumulus oocyte complexes were released from the oviducts and placed in pre-equilibrated fertilization drops consisting of HTF Media. Fresh sperm was isolated from the epididymis and vas deferens of male NOD/SCID mice (5 months of age) into HTF medium. Fertilization was carried out in HTF medium by transferring cumulus oocyte complexes to fertilization drops which contained the activated sperm and was incubated for 8 h at 37 °C (5% CO_2_). After incubation, the oocytes were washed in fresh HTF medium to remove excess sperm, incubation for 7 h at 37 °C (5% CO_2_) in M16 medium and microinjection performed in M2 medium.

### Microinjection and transfer to pseudopregnant recipients

Fertilized embryos with visible pronuclei following IVF were selected for microinjection and transferred to microinjection dishes containing M2 medium under mineral oil. The CRISPR/Cas9 reagent mixture was prepared by dilution of the components into DW to obtain the following concentrations: 40 ng/μl Cas9 protein (ToolGen, Korea) and 40 ng/μl IL2Rg single guide RNA (1, 2). The reagent mixture was introduced into the cytoplasm of fertilized embryos by microinjection using a continuous flow injection mode. Surviving two-cell stage embryos were surgically implanted into the oviducts of pseudopregnant female mice.

### Genotyping of the IL-2Rg deficient NOD/SCID mice

Genotypes of the pups born were analyzed using a PCR fragment that amplified the IL2Rg gene (primers used: FR 5′-CTTTGGCTCCGTCTCTCTGC-3′, RP 5′-TCCCTCTCAGGAGCTGTGTG-3′) by T7E1 assay (T7 endonuclease 1, ToolGen, Korea) and Sanger sequencing analysis (Bioneer, Korea). Homozygous deletion of 2 bp in the IL2Rg gene of NOD/SCID mice were maintained by mating homozygous mutant male mice with homozygous mutant female mice.

### Total RNA extraction and quantitative real-time PCR

Homozygous deletion of 2 bp in the background of NOD/SCID mice and NOD/SCID mice were analyzed for mRNA expression levels of IL2Rg. Total RNAs were prepared from mouse tail tissues using TRIzol (Molecular Research Center, USA), followed by cDNA synthesis from the total RNA samples using a first-strand cDNA synthesis kit (Fermentas, Canada). Real-time qRT-PCR assays were performed using a relative quantification protocol, the Exicycler™ 96 Real-Time Quantitative Thermal Block (Bioneer, Korea) and SYBR Premix Ex Taq (Takara, Japan). The following specific oligonucleotide primer sequences were used: IL2Rg_Tail, FP 5′-TCGAAGCTGGACGGAACTAA-3′, RP 5′- 187 CTCCGAACCCGAAATGTGTA-3′; IL2Rg_5’, FP 5′-188 CCTTCCAGAGGTTCAGTGCT-3′, RP 5′-ATAGTGCAGC 189 GTGAGGTTGG-3′; IL2Rg_middle’, FP 5′-TGCCTAGTGT 190 GGATGAGCTG-3′, RP 5′-CAGGCTGGCTCCATTTACTC-3′; 191 IL2Rg_3’, FP 5′-AACGAATGCCTCCAATTCC-3′, RP 5′- 192 TGGCAGAACCGTTCACTGTA-3′;GAPDH, FP 5′-AGA 193 ACATCATCCCTGCATGG-3′, RP 5′-CACATTGGGG 194 GTAGGAACAC-3′. All experiments were performed in triplicate.

### Analysis of serum in peripheral blood

Mice were fasted for 4 h after the indicated feeding regimen and were anesthetized using isoflurane for retro-orbital phlebotomy. Serum was prepared by spinning freshly collected blood in a cooled centrifuge at 1500 g for 10 min. The clear supernatant was collected and stored at − 70 °C until use. Serum AST, ALT, total cholesterol (CHO), triglyceride (TG), ALP and creatinine levels were determined using an auto analyzer (AU480 Chemistry System, USA).

### Xenograft

Tumorigenicities of HepG2, Raji and A549 cells were assayed by subcutaneous injection with 5 × 10^6^ or 1 × 10^6^ cells suspended in 100 μl matrigel, into the flanks of 8-week-old athymic nude, NOD/SCID and NIG(NSIG) male mice (*n* = 5 each). Solid tumor volume (in cubic millimeters) was determined by calipers and the formula of L × W2 × π/6, where L is the length and W is the width of the tumor; all tumors were photographed on the last day.

### Isolation of spleen cells from mice

To isolate the spleen cells, the harvested spleen was placed in a cell strainer, which was then positioned over a 4-well plate. Filtered medium (7 ml) was added and the spleen was pulverized. The strained, pulverized spleen cells were centrifuged at 1200 rpm for 5 min, and the supernatant was aspirated. Red blood cells (RBC) were removed by adding RBC lysis buffer (1 ml) to the pellet and allowed to incubate at room temperature for 10 min. The suspension cells were then mixed with FACS buffer (7 ml), centrifuged at 1200 rpm for 5 min, and the supernatant was discarded. The above procedure was repeated twice, and the final pellet was resuspended in 1 ml FACS buffer.

### Detection of T, B and NK-cell by flow cytometry

To measure the T, B and NK cell population, splenocytes were stained with fluorescein isothiocyanate (FITC)–conjugated anti-CD3, APC-conjugated anti-CD49b and phycoerythrin (PE)–Cy7–conjugated anti-B220 for 20 min at 4 °C. Stained cells were then washed with FACS buffer by centrifugation at 1200 rpm for 5 min. The cells were resuspended with Fix buffer and analyzed by flow cytometry on a Gallios flow cytometer (Beckman Coulter, USA).

## Results

### Generation of IL2Rg gene mutant NOD/SCID mice by CRISPR/Cas9 system

**Fig. 1:**
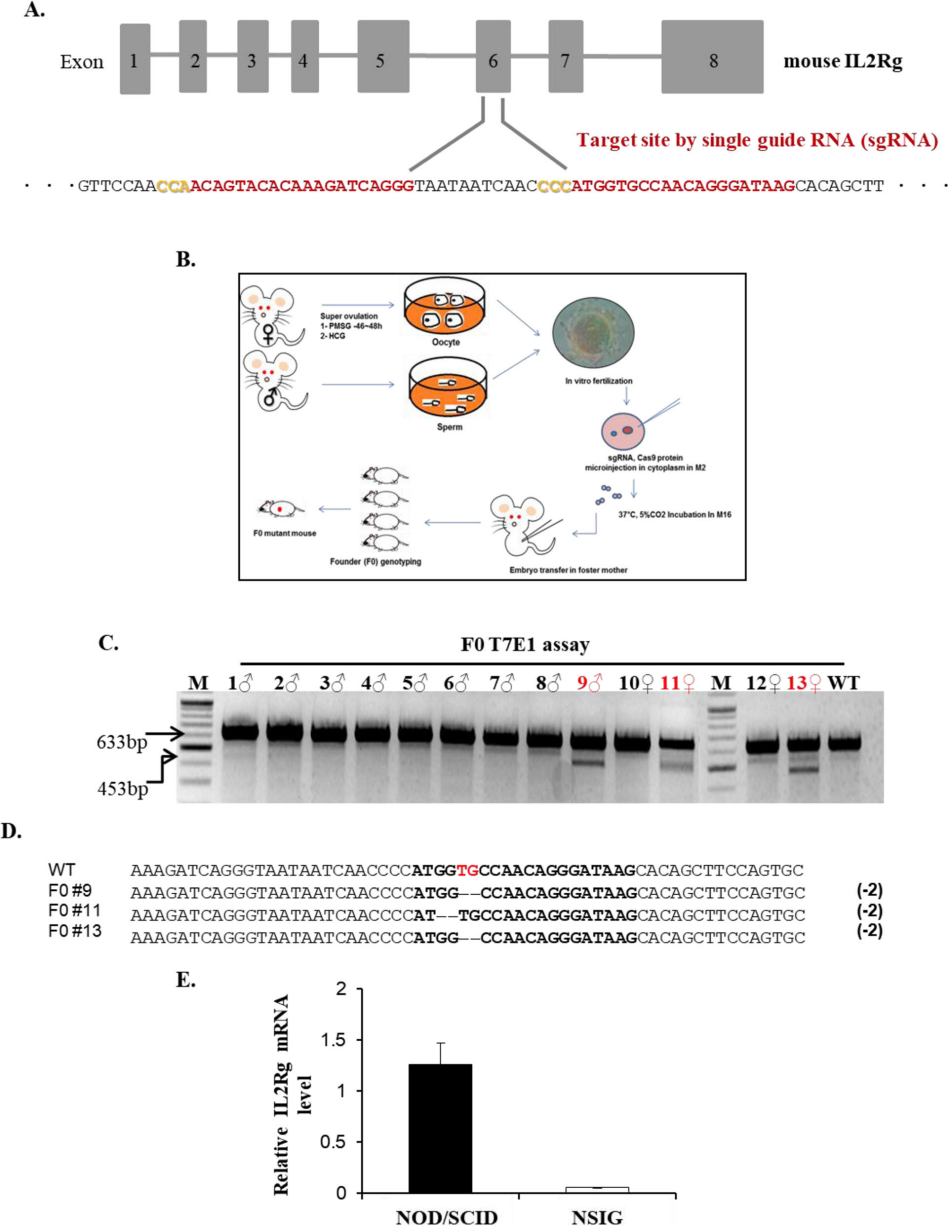
The generation of NIG(NSIG) mice by IVF and CRISPR/Cas9 system. **a** Schematic diagram of sgRNA targeting the mouse IL2Rg gene loci. The exon 1–8 region of the mouse IL2Rg gene is shown. The exon 6 sequence (upper case) are shown with 2 sgRNA sequences (labeled in red), and the PAM domain sequence NGG in yellow. **b** Schematic illustration of IVF and microinjection. Female NOD/SCID mice are super-ovulated with PMSG and hCG, followed by oocyte retrieval. Sperm is collected from male NOD/SCID mice. The oocytes and sperm are incubated to generate fertilized eggs and embryos, which are then microinjected with sgRNA and Cas9 protein. The injected embryos are transferred into pseudopregnant surrogate mothers (foster mothers). **c** Founder mice were genotyped by T7E1 assay after PCR amplification, and the T7E1 products were electrophoresed in 2.4% agarose gel. Mice were labeled #1–13, and M: marker, WT: wild type (negative control). WT allele is represented by a single band at 633 bp, while the heterozygous alleles (red labeling) are obtained as two bands at 453 bp and 633 bp. **d** Sequence analysis of the mutated IL2Rg alleles. The wild type sequence of exon 6 is shown on top. The sgRNA targeting sites are shown in bold letters. The mutant alleles of each mouse are labeled with the mouse ID number. The deleted sequences are marked in dash. **e** IL2Rg mRNA expression level results of F4 homo mutant mice. The mRNA levels of IL2Rg were determined by qRT-PCR analysis from mice tail tissue. Mice were labeled NOD.CB17/Prkdcscid/JKrb as NOD/SCID (positive control) and (−2 bp)^−/−^ (F4 homo mutant) mouse as NIG(NSIG)

The murine IL2Rg gene has 8 exons and encodes 266 amino acids. To induce IL2Rg gene mutation in NOD/SCID mice, we designed guide RNAs targeting the exon 6 region of IL2Rg gene (Fig. 1a). Before construction, we analyzed the off-target of two sgRNAs in IL2Rg using the NCBI blast system. Recent studies have shown that sgRNAs with mismatches of two or more nucleotides and other sequences do not have off-targets [23]. Off-target prediction shows that each sgRNA has rare off-target candidate sites (Table 1). The knockout of Exon 6 is predicted to encode a nonfunctional protein. In order to obtain IL2Rg-deficient mice, we performed microinjection of Cas9 and sgRNA into embryos from NOD/SCID mice.

**Table 1:** Off-target prediction for the sgRNAs (1 and 2) in IL2Rg

sgRNA	Target sequence	Exon	Strand	Off-targets			
				0	1	2	3
1	ACAGTACACAAAGATCAGGG	6	+	0	0	0	1
2	ATGGTGCCAACAGGGATAAG	6	+	0	0	0	0

A recent report suggests that it is difficult to obtain fertilized embryos after superovulation or natural mating in NOD/SCID mice [24]. Hence, since we were unable to obtain embryos by superovulation or natural mating, embryos for microinjection were generated by IVF from NOD/SCID mice. IVF is a widely used assisted reproductive technology, and is highly effective in producing healthy mice [21]. After identifying the fertilized embryos derived from IVF, the sgRNA (1, 2: 40 ng/ul) and Cas9 protein (40 ng/ul) were microinjected into the cytoplasm of fertilized embryos at the one cell stage, using a continuous flow injection mode. After appropriate incubation, surviving two cell embryos were implanted into the oviducts of pseudopregnant females (Fig. 1b and Table 2). Two pseudopregnant females gave birth to totally 13 mice pups. To analyze genotype of these pups, genomic DNA was extracted for PCR amplification, and the purified PCR products were then subjected to T7E1 digestion. Wild type allele is represented by a single band at 633 bp, while the heterozygous alleles (F0 mouse: 9, 11 and 13) are obtained as two bands at 633 and 453 bp (Fig. 1c). To define the mutations in the IL2Rg gene, the PCR products were subjected to DNA sequencing. Three mice had a 2 bp deletion at the sgRNA targeting site, while the other wild type had no modification (Fig. 1d). The PCR digestion and sequence results confirm that the F0 mice (9, 11 and 13) were heterozygous.

**Table 2:** IL2Rg knockout mice. 214 embryos were obtained from superovulated NOD/SCID female mice and 96 fertilized embryos were introduced with sgRNAs (1 and 2:40ng/ul) and Cas9 protein (40ng/ul). 45 embryos were survived and 38 embryos implanted into oviducts of two pseudopregnant female mice. 3 mutant mice were identified from 13 pups

Gene	Strain	No. of embryo				Newborns (Survived)	Mutant	Cas9 protein	sgRNA (1, 2)
		Collection	Injection	Survived	Transferred				
IL2Rg	NOD.CB17/Prkdcscid/JKrb	214	96	45	20	7	3	40 ng/ul	40 ng/ul
					18	6			

### Successful germline transmission of 2-bp deletion allele in the IL2Rg gene

To confirm whether the mutant genotypes are transmitted to next generation, we performed breeding by mating F0 male (#9) and female (#11, #13) with NOD/SCID female and male mice, respectively. The F0 #9 mouse bred F1 mice, but the other pair were unable to maintain their generation. To confirm transmission of the 2-bp deletion allele in the IL2Rg gene, we analyzed the genotype of F1 mice from F0 #9 mouse using the T7E1 assay and PCR product sequencing (Additional file 1). We confirmed the F1, F2 and F3 heterozygous mutant mice (Additional file 1); these mice were further mated with each other, thereby obtaining F4 homozygous mutant mice, which were then analyzed for their genotype using PCR product sequencing (Additional file 2). The homozygous mutant (−/−) mouse was classified as NIG(NSIG). To confirm the inability of the IL2Rg, RNAs were prepared from NOD/SCID (wild type) and NIG(NSIG) (F4 homozygous mutant) mice, and the mRNA levels of IL2Rg by qRT-PCR were subsequently determined. Our data indicates that the mRNA of IL2Rg in several tissue from NIG(NSIG) mice is not detected with background level (Fig. 1e and Additional file 3), thereby demonstrating that the 2-bp deletion allele of IL2Rg gene from F0 (#9) is germline transmitted and is null mutant allele as knockout mice.

### Basic characteristics of NIG mice

**Fig. 2:**
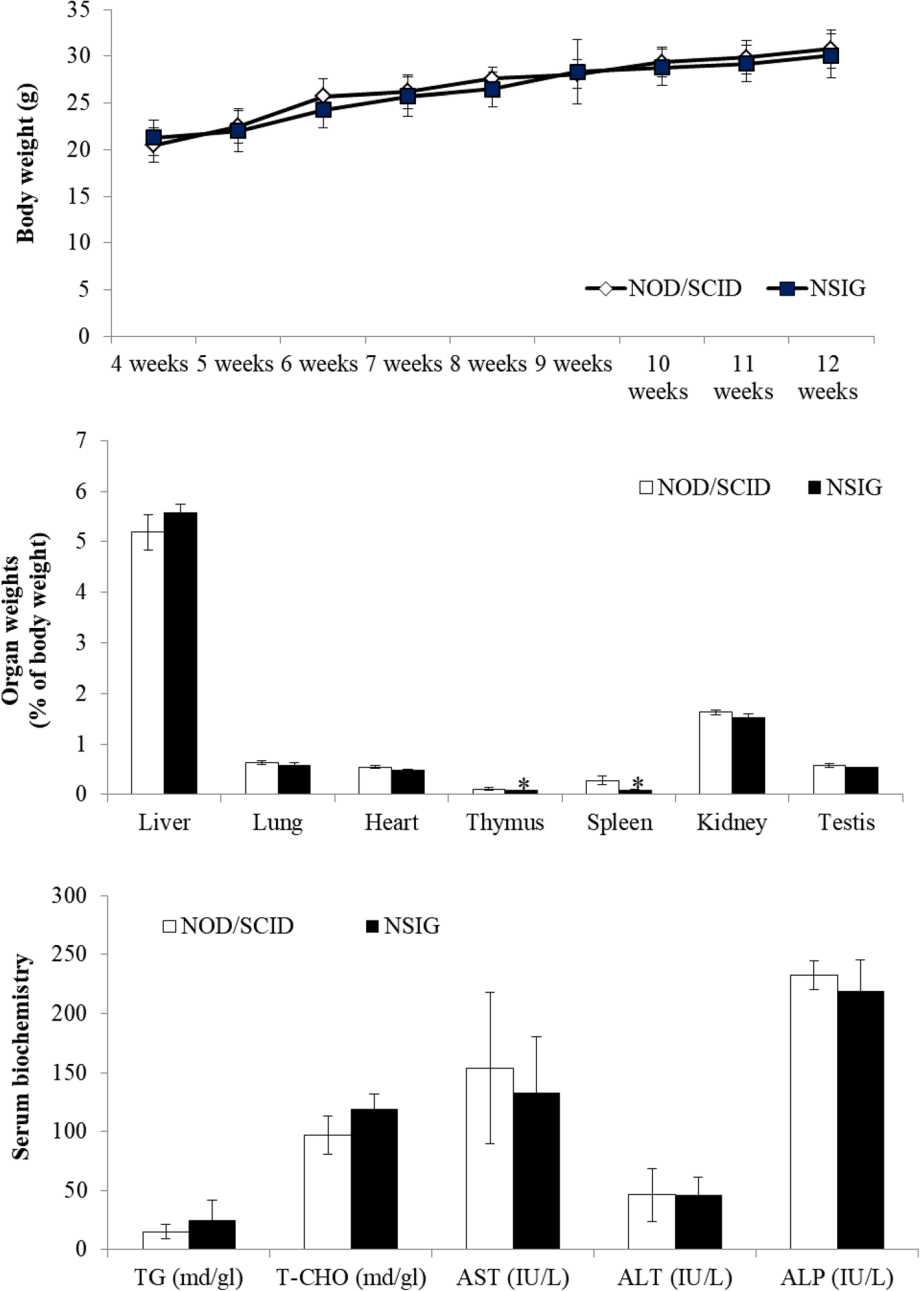
Analysis of basic characteristics in NIG(NSIG) mice. Mice were fed a normal diet for 13 weeks. **a** Body weight was measured weekly for 4 weeks until the end of the experiment (*n* = 4–8 in each group). **b** The weights for liver, thymus, lung, spleen, heart, kidney and testis were measured from the same mice as in A. **c** TG, T-CHO, AST, ALT and ALP levels were determined in serum from the same mice as in A. Data are presented as mean ± SD according to the Mann-Whitney U-test; *p < 0.05

To know basic phenotype of NIG mice, we measured the body and organ weights, and analyzed blood chemistry in serum collected from NOD/SCID and NIG(NSIG) mice (Fig. 2). We found that NIG(NSIG) mice had slightly decreased weight in thymus and spleen (Fig. 2b and Table 3) as compared to NOD/SCID mice. The weight loss of the thymus and spleen may be due to deficiency of T, B and NK cells. Although thymus and spleen size varied between the transgenic lines, morphologies of the tissues were normal (data not shown). Evaluation of hematologic parameters showed that no significant changes were observed in serological analysis of NOD/SCID and NIG(NSIG) mice (Fig. 2c, Table 4). These data indicate that NIG(NSIG) mice have no specific changes in body and organ weight, and in serological parameters excluding immune organ weights when compared to control mice.

**Table 3:** Organ weights

	Sex	No. of mice	Liver (g)	Thymus (g)	Lung (g)	Spleen (g)	Heart (g)	Kideny (g)	Testis (g)
NOD/SCID (13 weeks)	Male	4	1.11 (±0.15)	0.02 (±0.00)	0.13 (±0.02)	0.06 (±0.02)	0.11 (±0.00)	0.34 (±0.02)	0.12 (±0.01)
NIG (13 weeks)	Male	4	1.39 (±0.06)	0.02 (±0.00)	0.15 (±0.01)	0.02 (±0.04)	0.13 (±0.01)	0.39 (±0.02)	0.14 (±0.01)

**Table 4:** Serum biochemistry

	Sex	No. of mice	Creatinine	TG	AST	ALT	T-CHO	ALP
NOD/SCID (13 weeks)	Male	4	0.335	68.3	105.2	54.3	121.0	238.5
NIG (13 weeks)	Male	4	0.312	67.9	90.6	36.6	125.3	253.0

### Human cancer cell-line derived xenografts were successful in NIG mice without T&B and NK cells

**Fig. 3:**
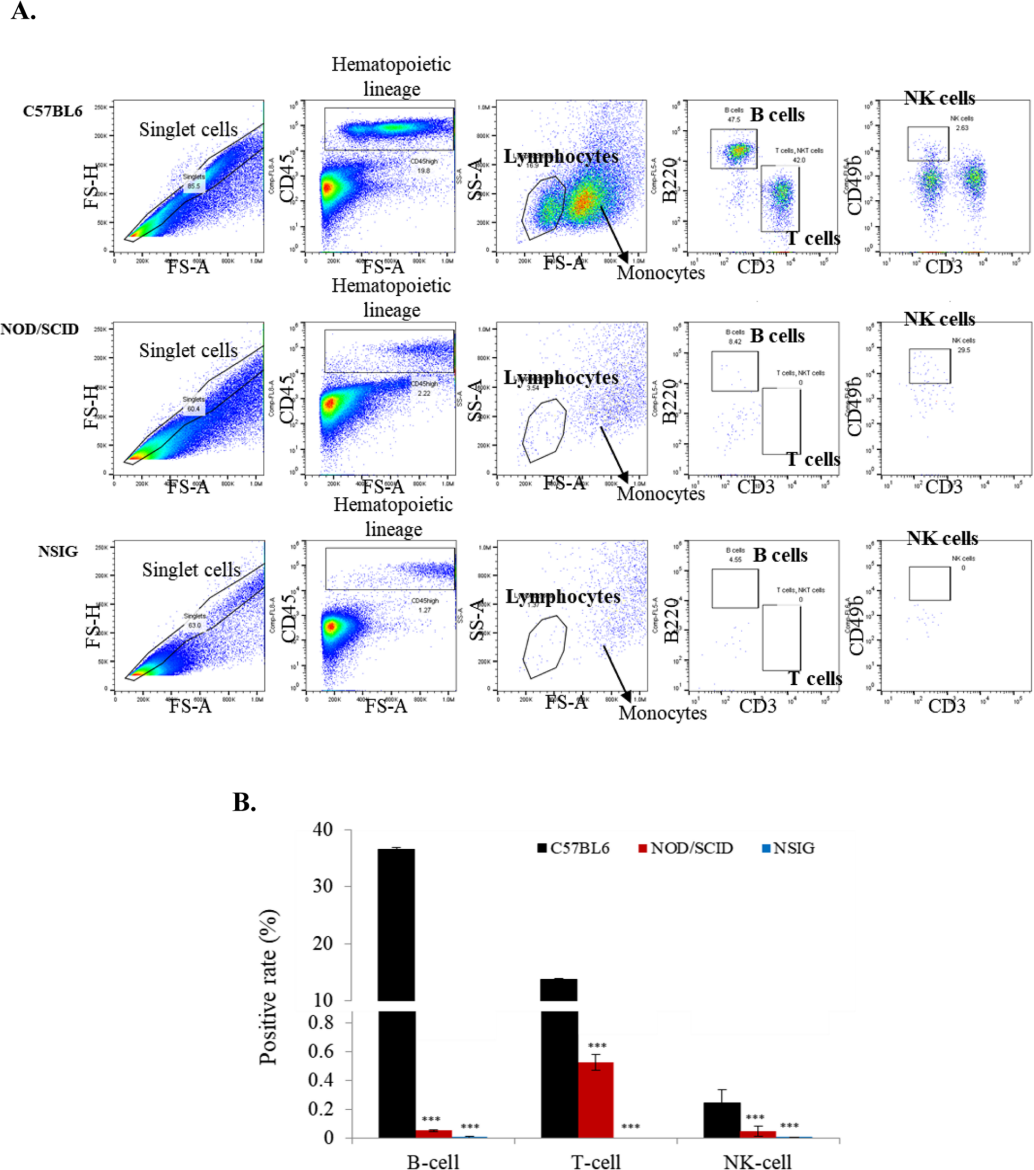
T, B, NK-Cell analysis in NIG(NSIG) mice. **a**, **b** Analysis of T, B and NK cell composition in spleen from C57BL/6 N, NOD/SCID and NIG(NSIG) male mice (*n* = 5 in each group). Data are presented as mean ± SD according to the Mann-Whitney U-test; *p < 0.05

**Fig. 4:**
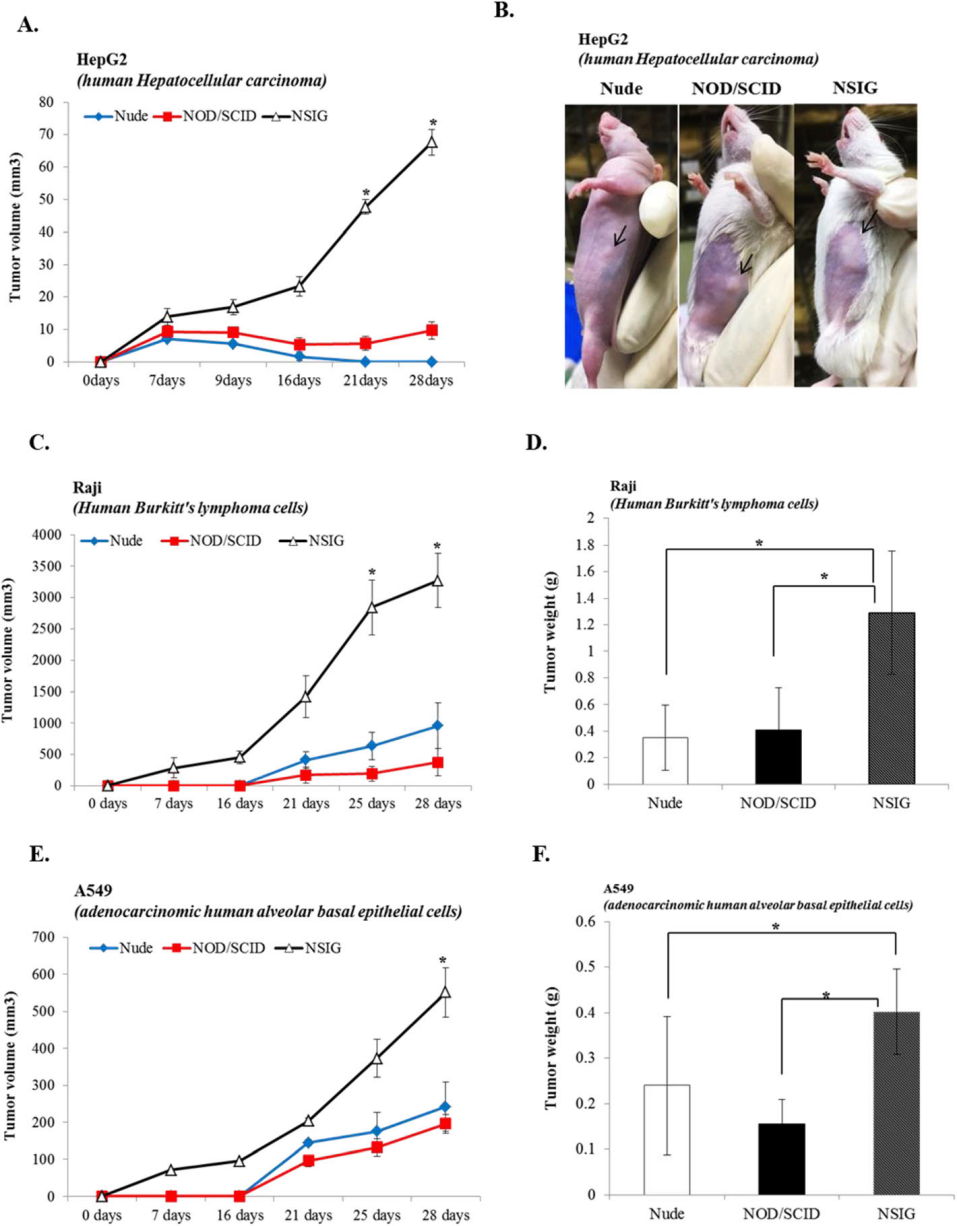
Human cancer cell-line derived xenograft in NIG(NSIG) mice. **a, c, e** The measurements of individual tumor lesion size in NIG(NSIG), NOD/SCID and nude male mice (n = 5 in each group). **b** Tumor view of HepG2 xenograft lesions in NIG(NSIG), NOD/SCID and nude mice. **d, f** Tumor weight of Raji and A549 xenograft in NIG(NSIG), NOD/SCID and nude mice. Data are presented as mean ± SD according to the Mann-Whitney U-test; *p < 0.05

We analyzed the lineage marker expression of T, B and NK cells in the splenocytes of NIG(NSIG) mice by flow cytometry, and compared the expression profiles with C57BL/6 N and NOD/SCID mice (Fig. 3a). NOD/SCID mice were deficient in T and B cells, as compared to C57BL/6 N, whereas NK cells were decreased relative to C57BL/6 N mice. NIG(NSIG) mice were devoid of T, B and NK cells (Fig. 3b). In addition, NIG(NSIG) mice show very similar composition of T, B and NK cells compared to NSG mice (Additional file 4). Previous studies have shown that the development of immunodeficient mice with mutations targeted at IL2Rg chain gene allows engraftment of the primary human tumor types [25]. Hence, to investigate whether NIG(NSIG) increases the xenograft effect on human cancer cell lines as compared to other mice, we performed an in vivo evaluation by subcutaneous injection of the human hepatocarcinoma cell line (HepG2), Human Burkitt’s lymphoma cells (Raji) and adenocarcinomic human alveolar basal epithelial cells (A549) in NIG(NSIG), NOD/SCID and nude mice, and measured tumor volumes one to two times weekly from 7 day after subcutaneous injection. We found highly increased tumor formation in the NIG(NSIG) mice, but no increase in tumor volume and weight was observed in other mouse groups (Fig. 4a-f). These results indicate that NIG(NSIG) mouse is a good model for xenograft of human cancer cells that are unable to form tumors in nude and NOD/SCID mice.

## Discussion

The mouse model has greatly contributed to advances in the field of immunology, especially after the foundation of inbred, transgenic, knock-out and knock-in mouse production systems [26, 27]. Though the results of immunology studies in mice cannot be simply extrapolated to humans, preclinical studies to predict the efficacy and safety of drugs have typically been performed using the rodent model [28]. Barriers of natural and acquired immune systems restrict human transplantation. Particularly, NK cell activity is an important obstacle for human cell and tissue engraftment [29]. NOD/SCID mice have lower levels of NK-cell activity [30]. To overcome these limitations the NOD/SCID model has been improved [15]. NSG and NOG mice were generated to eliminate NK cells [31, 32]. The IL2Rg acts to signal the IL-2, IL-4, IL-7, IL-9, IL-15, and IL-21 cytokines [11], and IL2Rg null mice have serious deficiencies in T, B and NK cell development. Immunodeficient mice, which are defined as humanized mice, have great advantages in the study of immunology [33, 34]. Immunodeficient mice are widely used for transplanting human normal and tumor cells [15], since they are capable of efficiently supporting engraftment of human hematopoietic stem cells [35, 36].

NOD/SCID mice present serious barriers towards introducing additional genetic modifications arising from the difficulty in deriving competent embryonic stem (ES) cell lines from NOD strain mice [17]. In other words, it is difficult to manipulate the NOD strain mice by embryo microinjection due to paucity of fertilized eggs obtained through natural mating [13, 37]. Therefore, transgenic or knockout mice with NOD genetic background, including NSG and NOG mice, were derived by backcrossing the NOD strain mouse with genetically modified mouse. Recent studies have reported that genetically modified mice have been successfully produced in NOD-derived immunodeficient mice by combining genome editing and IVF technologies [22]. In this study, the easy construction of sgRNA and high targeting efficiency of CRISPR/Cas9 system allowed for rapid generation of knockout mice by directly manipulating a small number of embryos via microinjection. Specifically, we used the IVF approach to obtain relatively more embryos for successful genetic modification by microinjection, and the CRISPR/Cas9 system that gave efficient genetic modification in NRG embryos, thereby enabling production of new mutant strain in the NRG mice within a few weeks. This method has several advantages, including a short time and cost saving in production of mutant mice, as compared with the ES cell derived gene targeting method.

To overcome previous limitations of the NOD background mice, we exploited both IVF and CRISPR/Cas9 genome editing technologies. We demonstrated that the IL2Rg knockout alleles were generated, and the germline could be transmitted in NOD/SCID mice. Also, we show that T and B cells were similarly deficient. In addition, NK cells were almost absent. Taken together, our results suggest that a small deletion of 2 bp in the IL2Rg gene of NOD/SCID mice by CRISPR/Cas9 system is sufficient to induce a severe immunodeficiency not available in nude and NOD/SCID mice, that could be used for cell engraftment of tumors.

Insertions and deletions (indels) emerge as the second most common type of human genetic variation, and a major cause of variation that accounts for the majority of species differences [38, 39]. Frame shift (FS) and non-frame shift (NFS) are two conversions caused by indels in the coding region. NFS indels comprise of three or more base pairs, indicating that one or more amino acid are inserted or deleted, with the remaining protein sequence remaining unchanged. On the other hand, FS indels shift the reading frame from the insert and delete position and can result in different protein sequences or early termination [25]. Recent genome sequencing projects have shown that indels contribute to the pathogenesis of diseases and changes in the expression levels of gene and protein functions [40]. Also, studies on the occurrence and locations of indels are important in understanding the origin of genetic variations [41]. Furthermore, recent studies have revealed that deletion and insertion of small fragments in human and mouse genes result in differences in the binding affinity and gene expression, which in turn lead to evolution and contribute to phenotypic diversity [25, 42]. Small deletions (− 2 bp deletion) in the IL2Rg gene causes premature termination of RNA transcription through frame shift mutation, thereby affecting the phenotype of mice.

## Conclusions

Taken together, we generated a severe immune deficient mouse NIG(NSIG) by small deletion of 2 bp in the IL2Rg gene in the background of NOD/SCID using genome editing in IVF-derived embryos. NIG(NSIG) may be a useful mouse model that can be exploited for xenograft of human cancer cells or tissues that are unable to form tumors in nude and NOD/SCID mice, as well as in the generation of humanized mice.

## Supplementary information

**Additional file 1.** Genotyping and sequencing of F1, F2 and F3 heterozygous mice. (A) Genotyping results of F1 generation mice. F1 mice were genotyped by the T7E1 assay with PCR amplificon, and resultant products were electrophoresed in 2.4% agarose gel. Wild type allele is represented by a single band at 633 bp, while the heterozygous alleles (red) are obtained as two bands at 453 bp and 633 bp. The sequence analysis of F1 heterozygous mice is shown in lower case. The target sites of sgRNAs are shown in bold letters. (B) PCR genotyping results of F2 and F3 generation mice. Wild type mouse genomic DNA serves as negative control. Mice were genotyped by PCR product sequencing with PCR amplificon. All mice are represented by a single band at 633 bp. (C) Sequence analysis of the heterozygous mutant alleles. The sequencing peak of the wild type mouse is shown in upper case and that of heterozygous mutant mouse in lower case. The mutant alleles of each mouse are labeled with the mouse ID number. The target sites of sgRNAs are shown in bold letters. The deleted sequences are marked in dash.

**Additional file 2.** Genotyping of F4 generation mice. The mouse numbers, from F4 #1–6, are shown above each lane. Wild type mouse genomic DNA serves as negative control. F4 mice were genotyped by PCR products sequencing with PCR amplificon. All mice are represented by a single band at 633 bp. (B) Sequence analysis of the mutated IL2Rg alleles. The sequencing peak of the wild type mouse is shown in upper case and that of F4 homozygous mutant mouse in lower case. The target sequence of sgRNAs are shown in bold letters. The mutant alleles of each mouse are labeled with the mouse ID number. The deleted sequences are marked in dash.

**Additional file 3. **Expression analysis of IL2Rg mRNA in lymphoid organs of NSIG mice. IL2Rg mRNA expression level results of NOD/SCID and NIG(NSIG) mice. The mRNA levels of IL2Rg were determined by qRT-PCR analysis in the three regions (5′, middle and 3′) of IL2Rg gene and RNAs were extracted from thymus and spleen tissues of NOD/SCID and NIG(NSIG) mice.

**Additional file 4.** T, B, NK cell analysis in NIG(NSIG) mice compared to NSG mice. (A) Analysis of T, B and NK cell composition in blood from C57BL/6, NIG(NSIG) and NSG male mice. (B) Analysis of T, B and NK cell composition in spleen from NIG(NSIG) and NSG male mice. (C) Graph quantifying results of (A) and (B).

## Data Availability

All data generated or analyzed during this study are included in this published article and its Additional files.

## Abbreviations

NK: Natural killer
IL2: Interleukin 2
IL2R: IL2 receptor
IL2Rg: Interleukin-2 receptor γ
ZFN: Zinc finger nuclease
TALENs: Transcription activator-like effector nucleases
GEM: Genetically engineered mice
DSB: Double-strand break
sgRNA: Single guide RNA
PAM: Protospacer adjacent motif
NHEJ: Error-prone non-homologous end-joining
IVF: In vitro fertilization
NOD/SCID: NOD.CB17/Prkdcscid/Jkrb mice
hCG: Human chorionic gonadotropin
CHO: Total cholesterol
TG: Triglyceride
NSG: NOD/ShiLtSz-scid/IL2Rγ null
NOG: NOD/ShiJic-scid/IL2Rγ null
indels: Insertions and deletions
FS: Frame shift
NFS: Non-frame shift
RBC: Red blood cell

## References

[1] Zhang L, Su L (2012). HIV-1 immunopathogenesis in humanized mouse models. Cell Mol Immunol.

[2] Brehm MA, Jouvet N, Greiner DL, Shultz LD (2013). Humanized mice for the study of infectious diseases. Curr Opin Immunol.

[3] Brehm MA, Shultz LD (2012). Human allograft rejection in humanized mice: a historical perspective. Cell Mol Immunol.

[4] Takebe T (2013). Vascularized and functional human liver from an iPSC-derived organ bud transplant. Nature.

[5] Zhu S (2014). Mouse liver repopulation with hepatocytes generated from human fibroblasts. Nature.

[6] Kitamura S, Sugihara K (2014). Current status of prediction of drug disposition and toxicity in humans using chimeric mice with humanized liver. Xenobiotica.

[7] Smith KA (1988). Interleukin-2: inception, impact, and implications. Science.

[8] Sugamura K, Asao H, Kondo M, Tanaka N, Ishii N, Nakamura M (1995). The common y chain for the multiple cytokine receptors. Adv Immunol.

[9] Kondo M, Obashi Y, Nakamura M, Sugamura K (1994). Expression of the mouse interleukin-2 receptor y chain in various cell popula- tions of the thymus and spleen. Eur J Immunol.

[10] Ishii N, Asao H, Kimura Y, Takeshita T, Nakamura M, Tsu-chiya S (1994). Impairment of ligand binding and growth signaling of mutant IL-2 receptor y - chains in patients with X-linked severe combined immunodeficiency. J Immunol.

[11] Sugamura K, Asao H, Kondo M, Tanaka N, Ishii N, Ohbo K (1996). The interleukin-2 receptor gamma chain: its role in the multiple cytokine receptor complexes and T cell development in XSCID. Annu Rev Immunol.

[12] Ohbo K, Suda T, Hashiyama M, Mantani A, Ikebe M, Miyakawa K (1996). Modulation of hematopoiesis in mice with a truncated mutant of the interleukin-2 receptor gamma chain. Blood.

[13] Cao X, Shores EW, Hu-Li J, Anver MR, Kelsall BL, Russell SM (1995). Defective lymphoid development in mice lacking expression of the common cytokine receptor gamma chain. Immunity.

[14] DiSanto JP, Müller W, Guy-Grand D, Fischer A, Rajewsky K (1995). Lymphoid development in mice with a targeted deletion of the interleukin 2 receptor gamma chain. Proc Natl Acad Sci U S A.

[15] Shultz LD, Ishikawa F, Greiner DL (2007). Humanized mice in translational biomedical research. Nat Rev Immunol.

[16] Machida K, Suemizu H, Kawai K, Ishikawa T, Sawada R, Ohnishi Y (2009). Higher susceptibility of NOG mice to xenotransplanted tumors. J Toxicol Sci.

[17] Nichols J, Jones K, Phillips JM, Newland SA, Roode M, Mansfield W (2009). Validated germline-competent embryonic stem cell lines from nonobese diabetic mice. Nat Med.

[18] Wang H, Yang H, Shivalila CS, Dawlaty MM, Cheng AW, Zhang F (2013). One-step generation of mice carrying mutations in multiple genes by CRISPR/Cas-mediated genome engineering. Cell.

[19] Zhou J, Wang J, Shen B, Chen L, Su Y, Yang J (2014). Dual sgRNAs facilitate CRISPR/Cas9-mediated mouse genome targeting. FEBS J.

[20] Fujii W, Onuma A, Sugiura K, Naito K (2014). One-step generation of phenotype-expressing triple-knockout mice with heritable mutated alleles by the CRISPR/Cas9 system. J Reprod Dev.

[21] Kohda T (2013). Effects of embryonic manipulation and epigenetics. J Hum Genet.

[22] Li F, Cowley DO, Banner D, Holle E, Zhang L, Su L (2014). Efficient genetic manipulation of the NOD-Rag1^−/−^IL2RgammaC-null mouse by combining in vitro fertilization and CRISPR/Cas9 technology. Sci Rep.

[23] Cho SW, Kim S, Kim Y, Kweon J, Kim HS, Bae S (2014). Analysis of off-target effects of CRISPR/Cas-derived RNA-guided endonucleases and nickases. Genome Res.

[24] Kumagai K, Kubota N, Saito TI, Sasako T, Takizawa R, Sudo K (2011). Generation of transgenic mice on an NOD/SCID background using the conventional microinjection technique. Biol Reprod.

[25] Lin M, Whitmire S, Chen J, Farrel A, Shi X, Guo J-t (2017). Effects of short indels on protein structure and function in human genomes. Sci Rep.

[26] Krimpenfort P, Rudenko G, Hochstenbach F, Guessow D, Berns A, Ploegh H (1987). Crosses of two independently derived transgenic mice demonstrate functional complementation of the genes encoding heavy (HLA-B27) and light (beta 2-microglobulin) chains of HLA class I antigens. EMBO J.

[27] Tepper RI, Levinson DA, Stanger BZ, Campos-Torres J, Abbas AK, Leder P (1990). IL-4 induces allergic-like inflammatory disease and alters T cell development in transgenic mice. Cell.

[28] Yamamoto T (2010). Animal model of systemic sclerosis. J Dermatol.

[29] Christianson SW, Greiner DL, Schweitzer IB, Gott B, Beamer GL, Schweitzer PA (1996). Role of natural killer cells on engraftment of human lymphoid cells and on metastasis of human T-lymphoblastoid leukemia cells in C57BL/6J-scid mice and in C57BL/6J-scid bg mice. Cell Immunol.

[30] Shultz LD, Schweitzer PA, Christianson SW, Gott B, Schweitzer IB, Tennent B (1995). Multiple defects in innate and adaptive immunologic function in NOD/LtSz-scid mice. J Immunol.

[31] Shultz LD, Lyons BL, Burzenski LM, Gott B, Chen X, Chaleff S (2005). Human lymphoid and myeloid cell development in NOD/LtSz-scid IL2R gamma null mice engrafted with mobilized human hemopoietic stem cells. J Immunol.

[32] Ito M, Hiramatsu H, Kobayashi K, Suzue K, Kawahata M, Hioki K (2002). NOD/SCID/gamma(c)(null) mouse: an excellent recipient mouse model for engraftment of human cells. Blood.

[33] Weissman A, Gotlieb L, Colgan T, Jurisicova A, Greenblatt EM, Casper RF (1999). Preliminary experience with subcutaneous human ovarian cortex transplantation in the NOD-SCID mouse. Biol Reprod.

[34] Suemizu H, Hasegawa M, Kawai K, Taniguchi K, Monnai M, Wakui M (2008). Establishment of a humanized model of liver using NOD/Shi-scid IL2Rgnull mice. Biochem Biophys Res Commun.

[35] Takenaka K, Prasolava TK, Wang JC, Mortin-Toth SM, Khalouei S, Gan OI (2007). Polymorphism in Sirpa modulates engraftment of human hematopoietic stem cells. Nat Immunol.

[36] Brehm MA, Cuthbert A, Yang C, Miller DM, DiIorio P, Laning J (2010). Parameters for establishing humanized mouse models to study human immunity: analysis of human hematopoietic stem cell engraftment in three immunodeficient strains of mice bearing the IL2rgamma(null) mutation. Clin Immunol.

[37] Auerbach AB, Norinsky R, Ho W, Losos K, Guo Q, Chatterjee S (2003). Strain-dependent differences in the efficiency of transgenic mouse production. Transgenic Res.

[38] Frazer KA, Chen X, Hinds DA, Pant PV, Patil N, Cox DR (2003). Genomic DNA insertions and deletions occur frequently between humans and nonhuman primates. Genome Res.

[39] Watanabe H, Fujiyama A, Hattori M, Taylor TD, Toyoda A, Kuroki Y (2004). DNA sequence and comparative analysis of chimpanzee chromosome 22. Nature.

[40] Dayi SU, Tartan Z, Terzi S, Kasikcioglu H, Uyarel H, Orhan G (2005). Influence of angiotensin converting enzyme insertion/deletion polymorphism on long-term total graft occlusion after coronary artery bypass surgery. Heart Surg Forum.

[41] Chen W, Zhang L (2015). The pattern of DNA cleavage intensity around indels. Sci Rep.

[42] Lyu Y-S, Shi P-l, Chen X-L, Zhao J, Gao X, Zhang X-N (2016). A small Indel mutant mouse model of Epidermolytic palmoplantar keratoderma and its application to mutant-specific shRNA therapy. Mol Ther Nucleic Acids.

