# Supplementary figures and images for: Two base pair deletion in IL2 receptor γ gene in NOD/SCID mice induces a highly severe immunodeficiency
Source: Lab Anim Res. 2020 Aug 14;36:27. doi: 10.1186/s42826-020-00048-y (PMC7427935; doi:10.1186/s42826-020-00048-y)

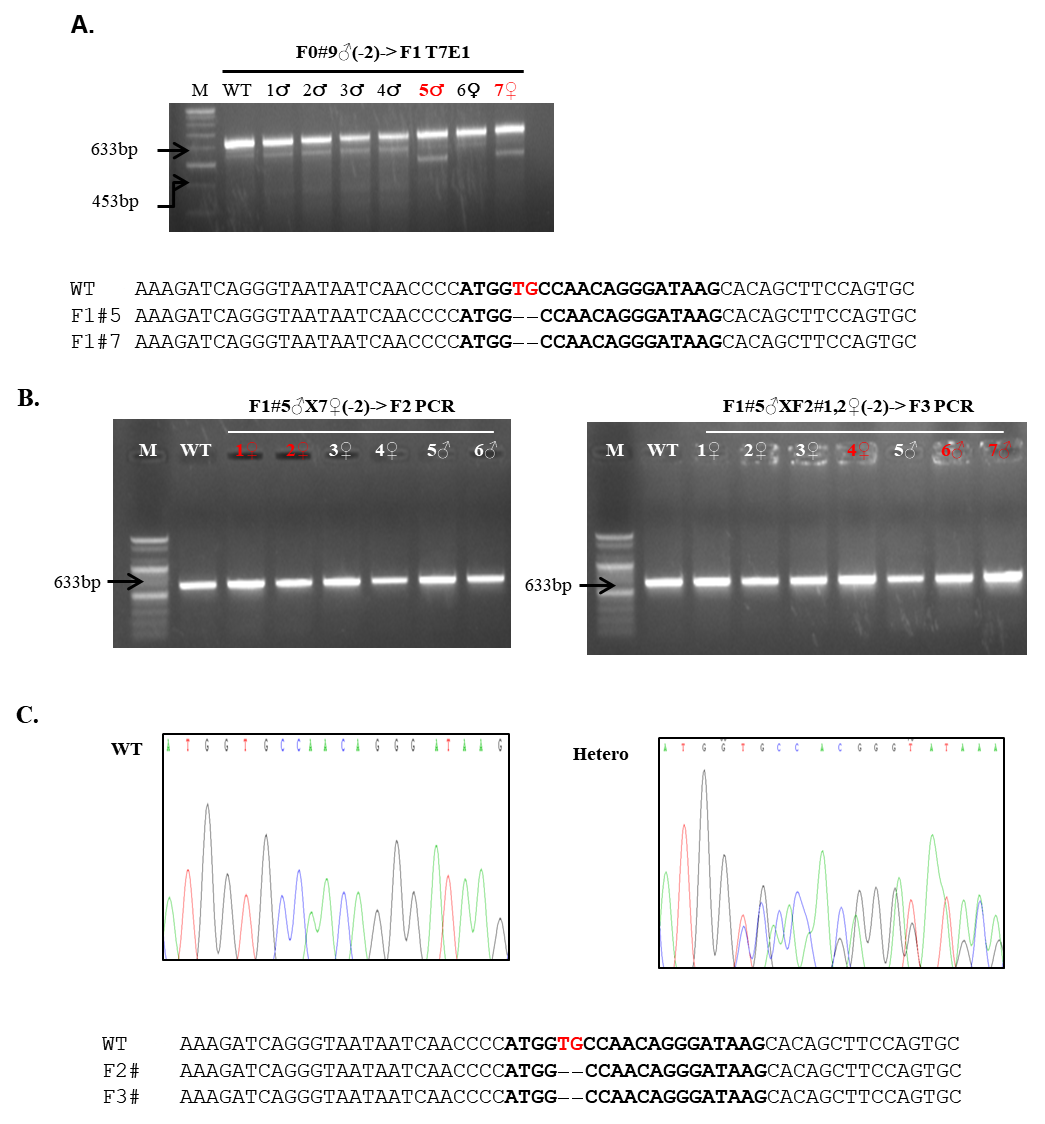


**Additional file 1. Genotyping and sequencing of F1, F2 and F3 hetero mice**

Supplement: Supplementary file 1 — Additional file 1. Genotyping and sequencing of F1, F2 and F3 heterozygous mice. (A) Genotyping results of F1 generation mice. F1 mice were genotyped by the T7E1 assay with PCR amplificon, and resultant products were electrophoresed in 2.4% agarose gel. Wild type allele is represented by a single band at 633 bp, while the heterozygous alleles (red) are obtained as two bands at 453 bp and 633 bp. The sequence analysis of F1 heterozygous mice is shown in lower case. The target sites of sgRNAs are shown in bold letters. (B) PCR genotyping results of F2 and F3 generation mice. Wild type mouse genomic DNA serves as negative control. Mice were genotyped by PCR product sequencing with PCR amplificon. All mice are represented by a single band at 633 bp. (C) Sequence analysis of the heterozygous mutant alleles. The sequencing peak of the wild type mouse is shown in upper case and that of heterozygous mutant mouse in lower case. The mutant alleles of each mouse are labeled with the mouse ID number. The target sites of sgRNAs are shown in bold letters. The deleted sequences are marked in dash. [file 42826_2020_48_MOESM1_ESM.docx]

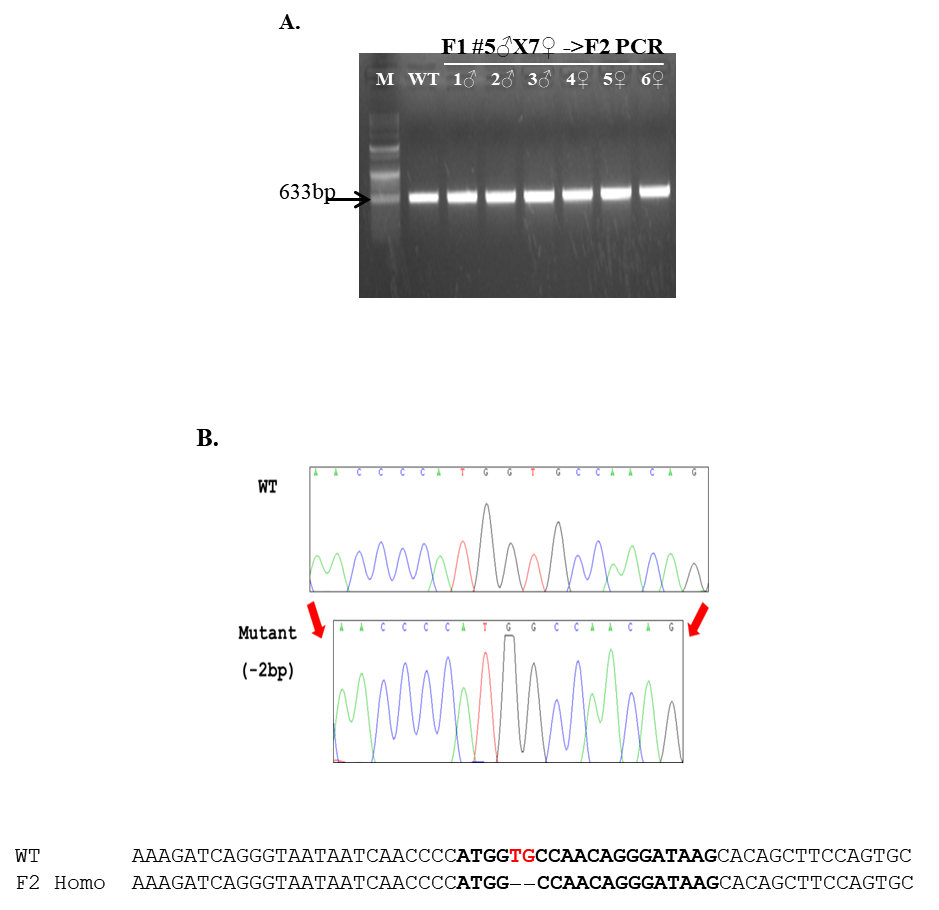


**Additional file 2. Genotyping and sequencing of F4 Homo mice**

Supplement: Supplementary file 2 — Additional file 2. Genotyping of F4 generation mice. The mouse numbers, from F4 #1–6, are shown above each lane. Wild type mouse genomic DNA serves as negative control. F4 mice were genotyped by PCR products sequencing with PCR amplificon. All mice are represented by a single band at 633 bp. (B) Sequence analysis of the mutated IL2Rg alleles. The sequencing peak of the wild type mouse is shown in upper case and that of F4 homozygous mutant mouse in lower case. The target sequence of sgRNAs are shown in bold letters. The mutant alleles of each mouse are labeled with the mouse ID number. The deleted sequences are marked in dash. [file 42826_2020_48_MOESM2_ESM.docx]

**
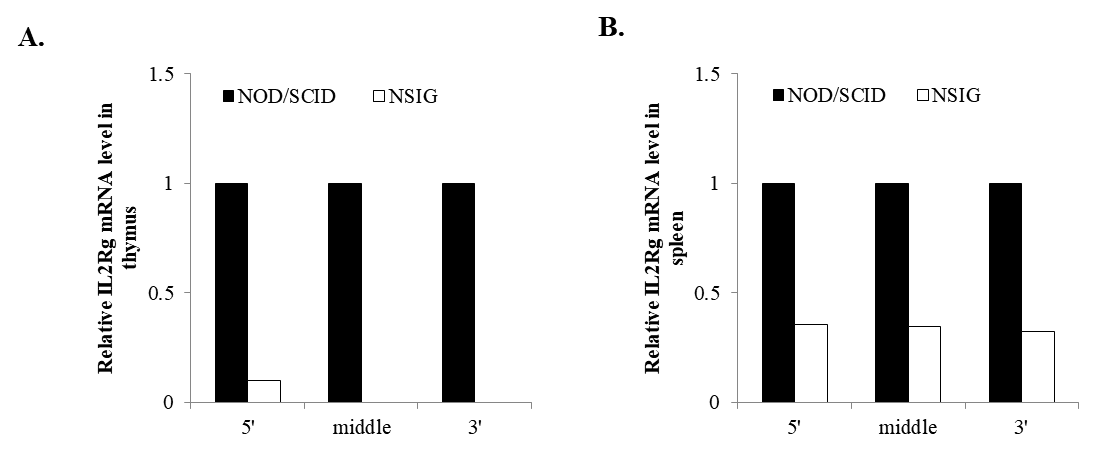
**

**Additional file 3. Expression analysis of IL2Rg mRNA in lymphoid organs of NSIG mice.**

Supplement: Supplementary file 3 — Additional file 3. Expression analysis of IL2Rg mRNA in lymphoid organs of NSIG mice. IL2Rg mRNA expression level results of NOD/SCID and NIG(NSIG) mice. The mRNA levels of IL2Rg were determined by qRT-PCR analysis in the three regions (5′, middle and 3′) of IL2Rg gene and RNAs were extracted from thymus and spleen tissues of NOD/SCID and NIG(NSIG) mice. [file 42826_2020_48_MOESM3_ESM.docx]

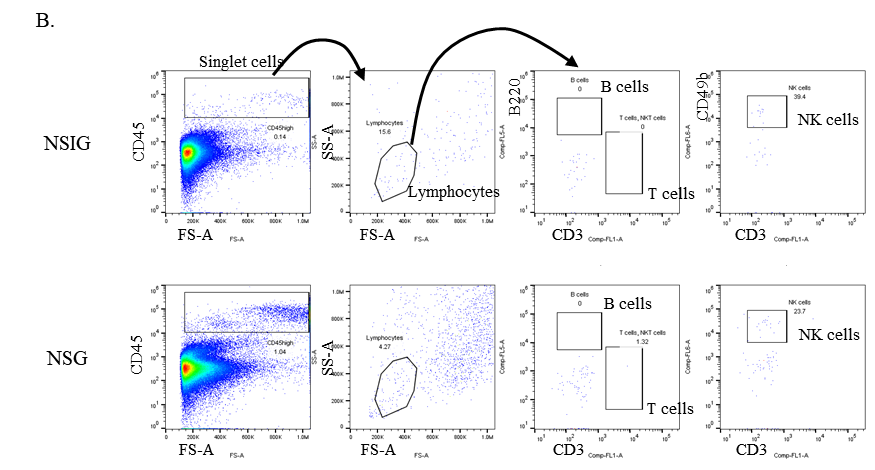

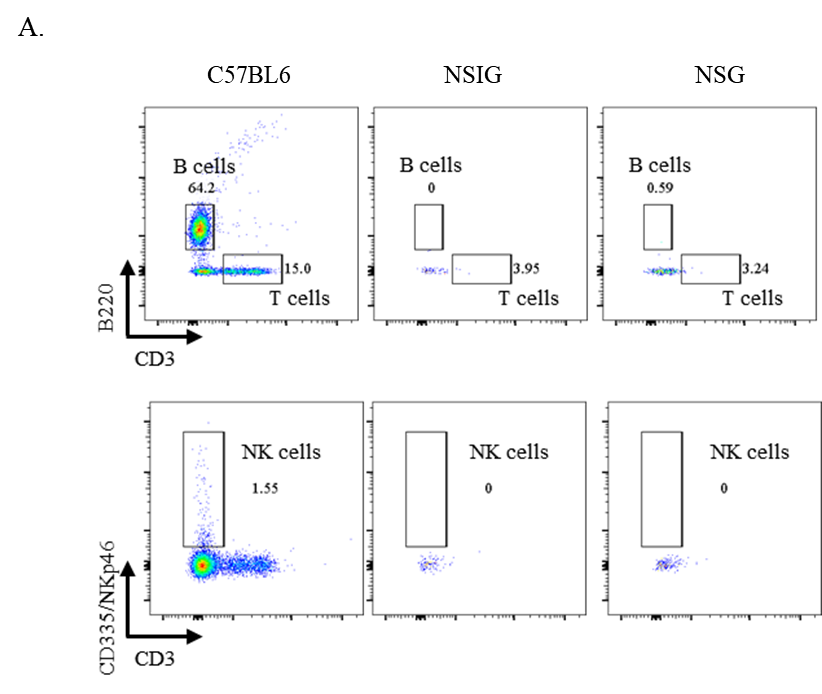

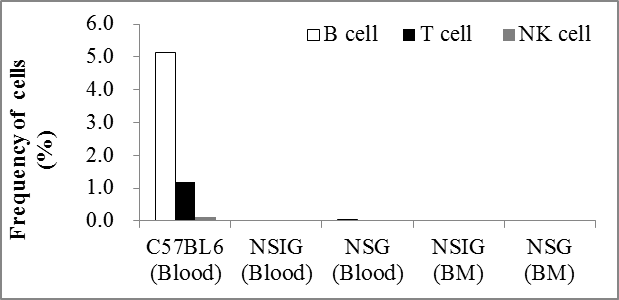


**Additional file 4. T, B, NK-Cell analysis in NSIG mice compared to NSG mice.**

Supplement: Supplementary file 4 — Additional file 4. T, B, NK cell analysis in NIG(NSIG) mice compared to NSG mice. (A) Analysis of T, B and NK cell composition in blood from C57BL/6, NIG(NSIG) and NSG male mice. (B) Analysis of T, B and NK cell composition in spleen from NIG(NSIG) and NSG male mice. (C) Graph quantifying results of (A) and (B). [file 42826_2020_48_MOESM4_ESM.docx]
